# A qualitative study on the experience of empowerment from the perspectives of breast cancer survivors

**DOI:** 10.1002/nop2.1000

**Published:** 2021-07-27

**Authors:** Zebing Luo, Chujun Chen, Wanzhu Xu, Peiru Wang, Yiru Wang

**Affiliations:** ^1^ Shantou University Medical College Shantou China; ^2^ Guangdong Provincial Key Laboratory of Breast Cancer Diagnosis and Treatment Shantou China; ^3^ Cancer Hospital of Shantou University Medical College Shantou China

**Keywords:** breast cancer survivors, empowerment, experience, interview, qualitative

## Abstract

**Aim:**

To describe the experience of empowerment for breast cancer survivors in order to increase quality of life after treatment.

**Design:**

We adopted a qualitative design in this study.

**Methods:**

A qualitative descriptive approach guided by interpretive description methodology as the theoretical framework was used in this study. Semi‐structured group and individual interviews were conducted with eleven female Chinese breast cancer survivors (mean age 54.18 years, 100% female).

**Results:**

Many survivors reported that they continued to experience disease‐related discomfort from the physiological aspect, but they use multiple ways to improve quality of life. Breast cancer survivors experienced empowerment after treatment. Empowerment mainly required three components: a belief in good health, capability of self‐management and acquisition of a good social support system.

## 1 INTRODUCTION

Breast cancer is the most prevalent cancer among females globally. According to statistics, there were ~2.1 million new breast cancer cases and ~63,000 breast cancer deaths in 2018 (Bray et al., 2018). Female breast cancer is also the most commonly diagnosed cancer worldwide, with 2.26 million cases having been diagnosed in 2020 (Ferlay et al., 2021). With the development of medical treatment, the 10‐year survival rates among breast cancer now exceeds 90%, which has contributed to a statistically significant number of breast cancer survivors (Akechi et al., 2018). The National Coalition for Cancer Survivorship of the United States regards cancer survivors as those who have undergone treatment after being diagnosed with cancer or those considered to be fully cured (Lee & Park, 2020). However, some reports about cancer survivors as those who have finished conventional therapy, such as surgery, radiotherapy and chemotherapy but includes those who have entered the follow‐up period or the endocrine treatment period (Mao & Sun, 2015). In this study, we use this latter definition. Modalities of treatments for breast cancer include surgery, chemotherapy, radiation, targeted therapy and hormone therapy (Wu & Zhao, 2020). After cancer treatment, some complications may persist for years, such as persistent pain (Juhl et al., 2016), breast cancer‐related lymphedema (Ferguson et al., 2016), and sleep disturbance (Singer et al., 2018). Among breast cancer survivors, the risk for lymphedema is 21% (Yeh et al., 2019), and the risk for sleep disturbance is 50% (Singer et al., 2018). Given the potential complications may lead to poor quality of life, the importance of health promotion has become statistically significant. Health promotion refers to the process of increasing the available options to individuals to get more control over their own health and environments (Mcqueen & De Salazar, 2011). Throughout the health promotion process, health empowerment is the key. Health empowerment theory refers to the process of helping individuals discover and explore their inner potential, at the same time, in order to achieve the goal of an individual being masters of his or her own health goals by emphasizing the integration of personal resources and social resources (Chen et al., 2018; Peng et al., 2020). The health empowerment theory suggests that empowerment is a well‐recognized predicting indicator of several patient outcomes, thus increasing empowerment of patients can indirectly improve their health outcomes in chronic diseases (Náfrádi et al., 2018). Furthermore, by promoting a patient's empowerment, patients can achieve a higher quality of life respond better to treatment, better manage and prevent complications, and develop positive and favourable attitudes towards the disease and life (Taleghani et al., 2014).

A previous study has reported that empowerment is important for cancer care and includes seven concepts: empowerment is an ongoing process, knowledge is power, has a positive role, communication and interaction between patients and healthcare personnel, group support, religion and spirituality and gender (Jørgensen et al., 2018). However, the connotation of health empowerment for Chinese older people is the process of initiating, performing and realizing health responsibly through the interaction of self, family and society (Zhang, 2012). Shabany pointed that the empowerment process of spinal cord injury included five components: patient's disruption in the existential integrity, injury recovery, inhibitors and facilitators of family‐centred empowerment and back on track (Shabany et al., 2020). Clearly, these studies point out that the process of empowerment is dynamic, complex, multifaceted and ongoing.

Identifying factors and processes that impact patient empowerment, which is a driver of patient‐centred care, is fundamental to achieve better patient outcomes. However, for different diseases that are difficult to diagnose and treat, the empowerment levels are different (Chiauzzi et al., 2016). Zhang (2012) constructed a health empowerment framework for elder Chinese patients with long‐term illness and Yazdkhasti et al., (2019) constructed an empowerment model for women to manage menopause. However, although several studies point out the importance of empowerment and the use of health empowerment on the increase, most empowerment strategies have mainly been used in the area of diabetes and few studies have investigated people with cancer empowerment (Liu, 2020). People with breast cancer think that during the disease period, empowerment is important and their empowerment needs to include information, beliefs and skills (Taleghani et al., 2014), but this study was conducted on people with breast cancer who were undergoing treatment. Therefore, the purpose of our study was to describe the experience of empowerment for breast cancer survivors in order to increase quality of life after treatment.

## 2 METHODS

### 2.1 Sampling

We used a widely performed interpretive, descriptive qualitative research approach, where the phenomenon of interest is qualitatively described to acquire information about patient experiences and deal with important clinical questions (Lin et al., 2020), to explore the experience of empowerment for breast cancer survivors. In October 2020, we conducted one pretested qualitative interview, one qualitative group interview and one qualitative individual interview. We used purposive sampling and maximum variation sampling to select breast cancer survivors who had different educational levels, different occupations and different treatment protocols. The ages of participants ranged from 42–65; the length of educational ranged from 6 years–16 years; the family income included low (<￥30,000), medium (￥30,000 ~￥60,000) and high (>￥60,000); types of insurance included employee medical insurance, community medical insurance and no health insurance; and 7 different treatment protocols were represented. The pretested interview was conducted in a hotel lounge, the group interview was conducted in a private room, and one individual interview was conducted by online video. All interviews were audio‐recorded, transcribed verbatim and analysed immediately after completion of all interviews.

Eleven participants were invited to participate in the study. All patients: (a) were female, diagnosed with breast cancer, and had completed the full treatment course, (b) were 18 years of age or older, (c) had undergone at least one treatment protocol. Exclusion criteria were: (a) cognitive/psychiatric impairment, (b) had recurrence or metastasis and (c) refusal to participate. Eleven females took part in this study. The age of the survivors ranged from 42–65 years (mean = 54.18, *SD *= 6.43). The survivors' characteristics are shown in Table 1.

**TABLE 1:** Demographic and clinical characteristics of study participants (*N* = 11)

Number	Age (year)	Education (year)	Employment	Family income^a^	Insurance	Religious belief	Prior treatment^b^
Pretest interview	42	16	Employed	High	Employee medical insurance	None	Surgery, CTX, RT
Survivor 1	58	9	Unemployed	Medium	Community medical insurance	None	Surgery, CTX, RT, TT, ET
Survivor 2	65	9	Unemployed	Medium	Community medical insurance	None	Surgery, CTX, TT
Survivor 3	56	6	Employed	Low	Community medical insurance	Buddhism	Surgery, CTX
Survivor 4	55	12	Self‐employed	High	Community medical insurance	None	Surgery, RT, ET
Survivor 5	53	12	Self‐employed	High	Community medical insurance	None	Surgery, CTX
Survivor 6	57	9	Employed	Low	Community medical insurance	None	Surgery, CTX, RT, TT
Survivor 7	58	9	Unemployed	High	Community medical insurance	Buddhism	Surgery, CTX
Survivor 8	53	12	Employed	Low	None	None	Surgery, CTX, TT
Survivor 9	44	6	Employed	Medium	Community medical insurance	Buddhism	Surgery, CTX, RT, ET
Survivor 10	55	12	Unemployed	Medium	Community medical insurance	None	Surgery, CTX, RT, ET

^a^ Family income (after tax): low <￥30,000, medium ￥30,000–￥60,000, high >￥60,000.

^b^ CTX = chemotherapy, RT = radiation therapy, TT = targeted therapy, ET = endocrinotherapy.

The study protocol was approved by the Ethics Committee of the Cancer Hospital of Shantou University Medical College (EC 2020053). Before each interview, the aims, procedures, potential risks and benefits of study were made clear to the participants, and participants had the right to withdraw at any time without any consequences. We promised that all participant information would be kept confidential and treated in confidence. All participants signed an informed consent document.

### 2.2 Data collection

Participants were recruited from August 2020–October 2020 by the responsible nursing researcher who through the electronic health record system, contacted eligible participants and assessed their interest via the mobile phone application WeChat. We contacted 15 breast cancer survivors but only 11 agreed to participate, 2 people said they had nothing to say, and 2 people said they did not have time to participate. The 11 participating breast cancer survivors had similar demographic characteristics, 6 people were 40–55 and 5 people were 56–70 years of age. 6 people had 0–9 years of education, and 5 people had an education of 10 years or more.

Our team consisted of 2 nursing postgraduate students, 1 head nurse and 1 nurse with several years' experience of nursing breast cancer. The interviews were performed by the primary researcher, a nursing postgraduate student who had experience in using qualitative research methods. The group interview was conducted in the local dialect, and the individual interview was conducted in the official Chinese language. Every interviews lasted between 30–50 min. Another researcher observed and documented each session. The moderator for the interviews built good relationships with the breast cancer survivors, and used standard interview techniques, such as pause, question, nod and induction. The assistant moderator was responsible for arrangement of the interview site, observed the group dynamics and gave the gifts. When data saturation was reached, data collection was stopped. The interview included two main questions: (1) What were the changes in your health‐promoting behaviours and state of mind after treatment? (2) What were the changes in your social relationships and working condition after treatment?

### 2.3 Data analysis

The focus group interview and individual interviews were audio‐recorded, translated into Chinese by the first author and checked by two researchers for accuracy. NVivo (Version 11) software was used to code all writing material. A qualitative content analysis approach was used in this study and involved a method of interpreting context through open coding classification of systems and identifying emerging themes (Hsieh & Shannon, 2005). Two nursing postgraduate students independently reviewed the content of each transcript using an open coding process in which that data was analysed line by line to identify words or phrases narrating empowerment experiences and then data content was assigned a code. These codes were used to identify patterns and recurrent themes. Data collection and analysis were performed in parallel. Thus, the open coding process was dynamic, and the classification of themes was constantly changing. Clusters that had similar content were grouped into themes and subthemes. When there was disagreement of open coding and classification, the corresponding author helped us to make a decision. When the answers of same aspect of breast cancer survivors had many similarities, and the data were saturated. At the same time, after having conducted the 11 interviews, we obtained the information about personal resources and social resources of breast cancer survivors after treatment. And after analysing the data, we obtained the information about the components of the empowerment phenomenon in breast cancer survivors and the relationships between these components.

## 3 RESULTS

Eleven breast cancer survivors took part in this study. Some survivors indicated that they experienced a difficult time, realized the preciousness of life and felt the beauty of life. They experienced empowerment after treatment, mainly with about to: (1) self‐empowerment, (2) family empowerment and (3) social empowerment. The analysis and connections between categories and themes are shown in Table 2.

**TABLE 2:** The analysis and connections between categories and themes

Themes	Categories	Examples
Self‐empowerment	Focus on physical health condition	(1) “Now I walk to work every day and increase my exercise in various ways” (pretest interview). (2) “Now, I go for a walk every morning and go to the pool every afternoon” (Survivor 1). (3) “After treatment, I would never make my affected limb to do too much work…and my diet consists of proper right nutrition, involving such as wolfberries, Codonopsis, and Astragalus” (Survivor 2). (4) ‘I exercise every day by imitating movement in instructional videos that were recorded by health care staff members” (Survivor 4). (5) “I elevate my affected limb every night” (Survivor 9). (6) “I try my best to avoid using my affected limb to bear gravity” (survivor 10).
	Alleviate symptom distress	(1) “I now more frequently perceive fatigue than before, so I avoid going to remote places as much as possible” (pretest interview). (2) “I have motion sickness after treatment, so I could hardly to take the car” (Survivor 3). (3) “My affected limb has lymphedema, and though I cannot cook, I think it can be overcome by relaxation” (Survivor 8). (4) “The main issue is lymphedema, and I use elastic sleeves and elevate my arm to relieve discomfort” (Survivor 10).
	Improve personal strength	(1) “We are not the most unlucky people…I should not be anxious, something which I cannot control will be decided by God… As long as we have confidence, an intense hurricane is always followed by a colourful rainbow” (pretest interview). (2) “a positive mindset is very important” (serious expression) (Survivor 2) (3) “I always go ask for blessings from Buddha” (Survivor 3). (4) “After my relatives knew my disease, they all treat me better than before” (Survivor 5). (5) “We should face and accept our impaired body, and learn to re‐love ourselves. It is a process of self‐acceptance” (Survivor 8). (6) “Because I am Buddhist, I often listen to Buddhist music, it can make me happy” (Survivor 9).
Family empowerment	Companion from family	(1) “My relatives are very kind to me, they did not look down upon me, and we always chatting and drinking tea” (Survivor 4). (2) “After my relatives knew my disease, they all treat me better than before” (Survivor 5). (3) “I always cook and drive my grandchildren to school every morning, and then exercise in the park. I cook and sleep at noon, then cook and pick up my grandchildren” (Survivor 7).
	Support from the spouse	(1) “My husband told me not to work in order to better recovery” (Survivor 1). (2) “My family and friends are particularly good to me, more than before, especially my husband (happiness on faces). My husband does all the housework (laughs), and treats me as a baby” (Survivor 5).
Social empowerment	Support from friend	(1) “My friends often visit me and help me with the house work, such as washing the dishes and wiping the table” (Survivor 3). (2) “My friends all know my illness, it is normal for their attitude towards me, we often chat together, and they did not segregate me deliberately” (Survivor 4) (3) “I am always singing and chatting together with my friends, and I am happy every day” (Survivor 5). (4) “I always sing with my friends during the weekend” (Survivor 6). (5) “I usually use the WeChat application to chat with my friends in my free time, so I don't feel lonely” (Survivor 10).
	Support from colleagues	(1) “My co‐workers are particularly nice. They always help me do my work secretly.” (pretest interview). (2) “My live is fulfilling, I go to square dancing every morning and work in pharmacies” (Survivor 4). (3) “Now I am working on the sales of health care products, I spend time with my colleagues every day and have a good time.” (Survivor 6).
	Support from healthcare staff	(1) “I started to implement a reasonable diet through the guidance of healthcare staff after treatment” (Survivor 6). (2) “The doctors and nurses were very kind to us” (Survivor 7). (3) “Fortunately, we still have doctors and nurses who love us so much, so we should cherish our second life” (Survivor 8).

### 3.1 Theme 1: Self‐empowerment

#### 3.1.1 Focus on physical health condition

Breast cancer survivors indicated that they paid more attention to their physical health conditions now than before treatment, but some survivors had also continued to experience sleep disturbances from the moment of their cancer diagnosis. Almost all survivors indicated that they tried their best to improve their physical health, such as by protecting the affected limb, reducing sedentary time, adopting a balanced diet, increasing the time of training and using traditional Chinese medicine. However, 2 survivors reported they were unable to overcome sleep disorder:

My smart watch will prompt me to exercise when I sit down for more than one hour… before my diagnosis of cancer, I took no more than 3,000 steps per day. Now I walk to work every day and increase my exercise in various ways… I was sleepless from the moment the doctors found a lump in my axillary lymph node (pretest interview).” “After treatment, I would never make my affected limb to do too much work…and my diet consists of proper right nutrition, involving such as wolfberries, Codonopsis, and Astragalus. (Survivor 2)

Diet and exercise play an important role in health‐promoting behaviours for breast cancer survivors:

Now, I go for a walk every morning and go to the pool every afternoon” (Survivor 1). I exercise every day by imitating movement in instructional videos that were recorded by healthcare staff. (Survivor 4)

Several survivors elevated the affected limb and avoided using the affected limb to bear gravity:

I elevate my affected limb every night. (Survivor 9). I try my best to avoid using my affected limb to bear gravity. (survivor 10)

Paying attention to the physical health can correct the survivor's previous bad habits and improve the body's ability to resist the disease.

#### 3.1.2 Alleviate symptom distress

More lymphedema and fatigue, rather than pain, were reported by survivors. They thought that symptoms were not worse after having undergone the dreadful disease, and some symptoms could be alleviated by certain methods:

I now more frequently perceive fatigue than before, and I will be too tired to ride more than half‐an‐hour, so I avoid going to remote places as much as possible. (pretest interview). I have motion sickness after treatment, so I could hardly to take the car. (Survivor 3). My affected limb has lymphedema, and though I cannot cook, I think it can be overcome by relaxation. (Survivor 8). My disease essentially has not impacted the quality of life after treatment. The main issue is lymphedema, and I use elastic sleeves and elevate my arm to relieve discomfort. (Survivor 10)

Symptoms are hard to avoid, but there are ways to reduce them and improve the quality of life of breast cancer survivors.

#### 3.1.3 Improve personal strength

Several survivors indicated that they employed multiple methods to improve personal strength, such as accepting their situation, having spiritual beliefs and finding the bright side of life:

We are unfortunate, but there are many the unlucky people in the world. We are not the most unlucky people…I should not be anxious, something which I cannot control will be decided by God. (pretest interview). We should face and accept our impaired body, and learn to re‐love ourselves. It is a process of self‐acceptance. (Survivor 8). I always go ask for blessings from Buddha. (Survivor 3). I like to do Buddhist exercise meditation. (Survivor 7). Because I am Buddhist, I often listen to Buddhist music, it can make me happy. (Survivor 9)

Over time, the survivors' mindsets were altered greatly and finding the bright side of life, with some survivors claiming that it was a chance for rebirth after having undergone this disease, and it was important for them to face life in a positive mindset:

After diagnosis, I thought it was very scary, and I was going to die immediately, but a year went by and I am alive and well. I want to talk to the breast cancer patients and tell them to not worry. As long as we have confidence, an intense hurricane is always followed by a colorful rainbow. (pretest interview). My family and friends cry when they see me. I told to them that I would not die. I have been alive for five years after completion of treatment, a positive mindset is very important (serious expression). (Survivor 2) Through experiencing this cancer, I felt that I was reborn. It was due to my having a positive mindset that I felt deeply that good comes from bad. (Survivor 8)

Self their situation, becoming spiritual and finding the bright side of life was a difficult process, but in the end, they found a rainbow.

### 3.2 Theme 2: Family empowerment

#### 3.2.1 Companion from family

The company of family members was helpful for breast cancer survivors to establish a rich life:

My relatives are very kind to me, they did not look down upon me, and we always chatting and drinking tea. (Survivor 4). After my relatives knew my disease, they all treat me better than before. (Survivor 5). I always cook and drive my grandchildren to school every morning, and then exercise in the park. I cook and sleep at noon, then cook and pick up my grandchildren. (Survivor 7)

The company of family members was rarely mentioned in this study. This may be because most of the family members bore the financial burden of the family and had to work, so they had little time to accompany of breast cancer survivors.

#### 3.2.2 Support from the spouse

All breast cancer survivors in this study were married. Support from spouse was very important for the survivor, and it did not matter how old the survivor was. Once the patient was diagnosed, the burden of the family was basically placed on the spouse, and although breast cancer survivors had recovered, some of them remain the status of being cared for by the family:

My husband told me not to work in order to better recovery. (Survivor 1). My family and friends are particularly good to me, more than before, especially my husband (happiness on faces). My husband does all the housework (laughs), and treats me as a baby. (Survivor 5)

The support from spouse brings them happiness.

### 3.3 Theme 3: Social empowerment

#### 3.3.1 Support from friend

“Friends” was mentioned many times. Friends occupied an important place in the hearts of some breast cancer survivors:

My friends often visit me and help me with the house work, such as washing the dishes and wiping the table. (Survivor 3). My friends all know my illness, it is normal for their attitude towards me, we often chat together, and they did not segregate me deliberately. (Survivor 4) I am always singing and chatting together with my friends, and I am happy every day. (Survivor 5). I always sing with my friends during the weekend. (Survivor 6). I usually use the WeChat application to chat with my friends in my free time, so I don't feel lonely. (Survivor 10)

True friends are there when someone is needed, and true friends can help breast cancer survivors to better integrate into social interactions.

#### 3.3.2 Support from colleagues

Working can help the survivor get the support from colleagues during working, and reintegrate into family and society more quickly. In this study, seven survivors had selected to work in order to fulfil their lives. Three survivors refused to work again because of age and physical condition:

My co‐workers are particularly nice. They always help me do my work secretly. (pretest interview). My live is fulfilling, I go to square dancing every morning and work in pharmacies. (Survivor 4). Now I am working on the sales of health care products, I spend time with my colleagues every day and have a good time. (Survivor 6)

Thus, a good job and working environment can help breast cancer survivors gain more empowerment.

#### 3.3.3 Support from healthcare staff members

The support from healthcare staff members can make breast cancer survivors feel care and love, and improve their confidence in recovery:

I started to implement a reasonable diet through the guidance of healthcare staff after treatment. (Survivor 6) The doctors and nurses were very kind to us. (Survivor 7) Fortunately, we still have doctors and nurses who love us so much, so we should cherish our second life. (Survivor 8)

The support from healthcare staff members plays an important role in the recovery of breast cancer survivors after treatment.

## 4 DISCUSSION

This study shows the experience of empowerment of the 11 breast cancer survivors, which principally involved self‐empowerment, family empowerment and social empowerment. Another study, conducted in Mexico, pointed out that after completing primary cancer treatment, on the one hand, cancer survivors receive poor physical, emotional and social experiences, such as adverse physical and sexual experiences, emotional problems, cancer‐related stigma; and on the other hand, had difficulty in acquiring health‐related information and economic help, as well as experienced distress in facing their body image and interacting with support groups (Knaul, 2020). On the contrary, we find that the breast cancer survivors will focus on physical health, alleviate symptom distress, improve personal strength and receive support from family and society. This may be because of the selection of research patients. In Knaul's study, breast cancer survivors, cervical cancer survivors and prostate cancer survivors were included (Knaul, 2020), but in our study, only breast cancer survivors were included. In common with Knaul's study is the emphasis on family empowerment and belief that their future depends on God. At the same time, support from the spouse is emphasized in our study. As Regan said, patients seek to cope with the disease as a couple, including sharing emotions, getting support and overcoming cancer‐related problems together (Regan et al., 2015). In addition, the level of empowerment may also be related to the level of economics. A study of rural African‐American breast cancer survivors on their survivorship experiences had pointed out that they lack knowledge about survivorship, education and support, but they also believed that spirituality and religion are the key to coping and accepting cancer (Adams et al., 2017).

It is obvious that many cancer survivors have to cope with some difficulty after completing primary cancer treatment, but when facing difficulties, cancer survivors in different countries may have different experiences. The process of empowerment promotes and strengthens the capacity of people to meet their needs and solve problems. A previous study also reported the positive effects of empowerment, particularly with regard to improved resilience and posttraumatic growth levels (Üzar‐Özçetin & Hiçdurmaz, 2019). Thus, the effect of empowerment may enable cancer survivors to enjoy life after treatment, and better return to family and society and medical care staff members should ensure policies are implemented to improve the level of empowerment of breast cancer survivors.

The World Health Organization (2006) reported that empowerment is a strategy that includes positive co‐operative relationships and patient self‐care in order to improve the healthy outcome and quality of life of patients with chronic diseases. Health empowerment theory advocates that the patient's drive for establishing healthy behaviours should be increased, in order to improve the patient's ability to control his or her environment and life (Liu et al., 2020). As one of our participants said, it is a process of re‐loving themselves, and almost all breast cancer survivors try to do something in order to improve their health. Similar to our study, Rashidi et al. reported that people with breast cancer have to undergo a process of “identity destruction” and “identity reconstruction” and finally become empowered (Rashidi et al., 2020).

According to reports, approximately 90% of breast cancer survivors may experience long‐term complications following treatment, which might lead to psychological disorders, a terrible body image and lower quality of life (Lovelace et al., 2019). With the development of health care, cancer has become a long‐term illness, and improvement of self‐management capability that includes treatment, lifestyle modifications, and biopsychosocial‐spiritual consequences of diseases is important for living with cancer (Baydoun et al., 2018). So, the period after diagnosis of cancer is an important time for people with breast cancer to re‐evaluate their top priorities and make greater effort towards a healthier lifestyle in order to improve health (Ganz & Goodwin, 2015). Likewise, for breast cancer survivors, it is important for them to maintain overall health, and adopting a secondary prevention strategy, that consists of paying more attention to healthy lifestyles and correctly treating non‐breast cancer‐related health problems, should be a top priority (Ganz & Goodwin, 2015). By avoiding and preventing long‐term complications, breast cancer survivors will become more self‐empowered.

In this study, some breast cancer survivors reported that they have some physical discomfort, including lymphedema, fatigue, motion sickness and sleep disorder, but most of them were able to alleviate the symptoms by multiple means, such as relaxation, exercise, eating a balanced diet, reading, protecting the affected limb and avoiding sedentary time. With regular follow‐up, cancer‐related toxicities can be better managed, therapy adherence and a healthy lifestyle can be improved, and psychosocial support can be given (Ruddy et al., 2020). Many guidelines, such as the American Cancer Society, American Society of Clinical Oncology and National Comprehensive Cancer Network, recommend that breast cancer survivors have a history and physical examination at least once a year. However, for 69.6% of breast cancer survivors the mean time for follow‐up is three years (Ruddy et al., 2020). Future research should pay more attention to increasing follow‐up compliance, offer targeted preventive care and treat adverse effects. Long‐term effects of breast cancer include physical, functional, emotional and psychosocial changes (Lovelace et al., 2019). Carter et al. reported that physical activity training programmes that included 6 weeks of supervised exercise sessions and 6 weeks of home‐based workouts can lower physiological stress and significantly improve fatigue (Carter et al., 2016). A meta‐analysis (Hasenoehrl et al., 2020) pointed out that it was resistance exercise that appeared to decrease the risk of lymphedema and improve upper and lower extremity strength for people with breast cancer, but more evidence is needed for the role of resistance exercise in ameliorating lymphedema in people with breast cancer. All in all, focussing on physical activity can improve the physical condition and decrease the discomfort of breast cancer survivors.

Psychosocial support is also important for breast cancer survivors and can help them to weather difficult times. In this study, our participants may adopt some ways to overcome difficulties, such as improve personal strength. Some may acquire family empowerment and social empowerment, such as through working with or having a family companion, or receiving support from the spouse, friends, colleagues and healthcare staff members. Good interpersonal communication can improve the survivor's ability to adapt to society and encourage them to open their hearts and share their joys and sorrows with those around them.

However, the psychosocial support needs of people with cancer are underappreciated, possibly because of a lack of multidisciplinary teamwork, difficulties in physician–patient communication, and problems in palliative care settings (Steven et al., 2019). As Wang et al. reported, family support plays an important role for Chinese breast cancer survivors during the treatment time (Wang et al., 2020), and social support is important for people with breast cancer during the vulnerable period (Zhang et al., 2018), which is similar to our study, but our study further emphasizes the importance of family empowerment and social empowerment, which healthcare staff members should ensure for breast cancer survivors.

## 5 IMPLICATIONS AND RECOMMENDATIONS

This study describes that the experience of empowerment for breast cancer survivors involves three components: self‐empowerment, family empowerment and social empowerment. Understanding the experience of empowerment with the breast cancer survivors can help clinical nursing staff members to benefit most survivors with breast cancer and help them to better integrate into family and society. In addition, healthcare staff members should understand and implement the means of empowerment. For some survivors who have negative views of the future, healthcare staff members can help them during the most vulnerable period to change the negative attitude. Furthermore, healthcare staff members should pay more attention to survivors who have completed treatment, as well as meet the individual needs for care. Further research needs to focus on and develop measures to improve the empowerment of breast cancer survivors. In addition, exploring factors related to empowerment among breast cancer survivors would help to develop more individual and effective interventions.

## 6 LIMITATIONS OF THE STUDY

Although we can more thoroughly understand the experience of empowerment with the breast cancer survivors, this research has some shortcomings. Firstly, our study sample size is small, and future studies should involve a larger sample size. Secondly, our participants come from only one oncology specialty hospital, so multicentre studies are required in the future. Finally, we only interview Chinese, so the findings may not be generalizable to other countries because cancer survivors in different countries may have different experiences of empowerment.

## 7 CONCLUSIONS

In this study, many breast cancer survivors report that they continue to experience physical, disease‐related discomfort, but use multiple ways to improve quality of life, such as focussing on and adopting a healthy lifestyle. In terms of psychological state, survivors learn to improve personal strength, some with the help of spiritual beliefs. In terms of social conditions, survivors have crucial support from family, friends, colleagues and healthcare staff members. These findings enable a better understanding of the experience of empowerment from the perspectives of breast cancer survivors to better help nurses to give out‐of‐hospital care for breast cancer survivors.

## CONFLICTS OF INTEREST

The authors have no conflicts of interest to disclose.

## AUTHOR CONTRIBUTIONS

Zebing Luo and Chunjun Chen contributed to the conception of the study. Zebing Luo, Wanzhu Xu and Peiru Wang contributed significantly to collect and analyse the data. Yiru Wang contributed significantly to guide the study.

## Data Availability

The data that support the findings of this study are available on request from the corresponding author. The data are not publicly available due to privacy or ethical restrictions.
